# 1434. Incidence of Tuberculosis after Transplantation: A Nationwide Cohort Study in South Korea, 2008-2020

**DOI:** 10.1093/ofid/ofac492.1263

**Published:** 2022-12-15

**Authors:** JongHoon Hyun, Su Jin Jeong, Ji-Man Kang

**Affiliations:** Inje University Ilsan Paik Hospital, Goyang, Kyonggi-do, Republic of Korea; Yonsei University College of Medicine, Seoul, Seoul-t'ukpyolsi, Republic of Korea; Severance Children’s Hospital, Yonsei University College of Medicine, Seoul, Seoul-t'ukpyolsi, Republic of Korea

## Abstract

**Background:**

Tuberculosis (TB) is one of the leading causes of morbidity and mortality among transplant recipient. The purpose of this study is to evaluate the standard incidence ratios (SIRs) of TB in all transplant recipient and to compare which is more risk by transplant type.

**Methods:**

We conducted a nationwide population-based retrospective study using data retrieved from the Health Insurance Review and Assessment Service. Active TB case were studied, and SIRs was calculated based on the most recent year, 2019. SIRs were computed as the ratio of observed to expected numbers of TB, based on national age and sex. All analyses were conducted by transplant type and age group.

**Results:**

From 2008 to 2020, a total of 57,103 patients who underwent transplantation were included. 830 cases were confirmed with TB (1.45%) and median time to develop TB was 1.74 year following transplantation. When analyzing the cumulative incidence by transplant group, it was in the order of allogenic hematopoietic stem cell transplantation (HSCT), solid organ transplantation (SOT), and autologous HSCT (*P*< 0.001). In SOT, liver transplantation had the highest incidence of TB, followed by lung, heart, and kidney (*P*< 0.001). The cumulative incidence for the age group increased with age (*P*< 0.001). Incidence of TB was markedly increased among transplant recipients compared with general population (SIR: 3.53, Confidence interval (CI): 3.38-3.67). SIR varied according to age group: age under 20 years (SIR: 4.98 CI: 3.16-7.47), age between 20 and 40 years (SIR: 5.83 CI: 5.22-6.49), age between 40 and 60 years (SIR: 4.66 CI: 4.42-4.92), age over 60 years (SIR: 2.05 CI: 1.89-2.21). Based on a multivariate analysis, male sex (hazard ratio (HR) = 1.398; *P* < 0.001), previous TB history (HR = 1.515; *P*=0.014) were the common independent risk factors for TB after transplantation.

Baseline characteristics of study population

**Figure:**
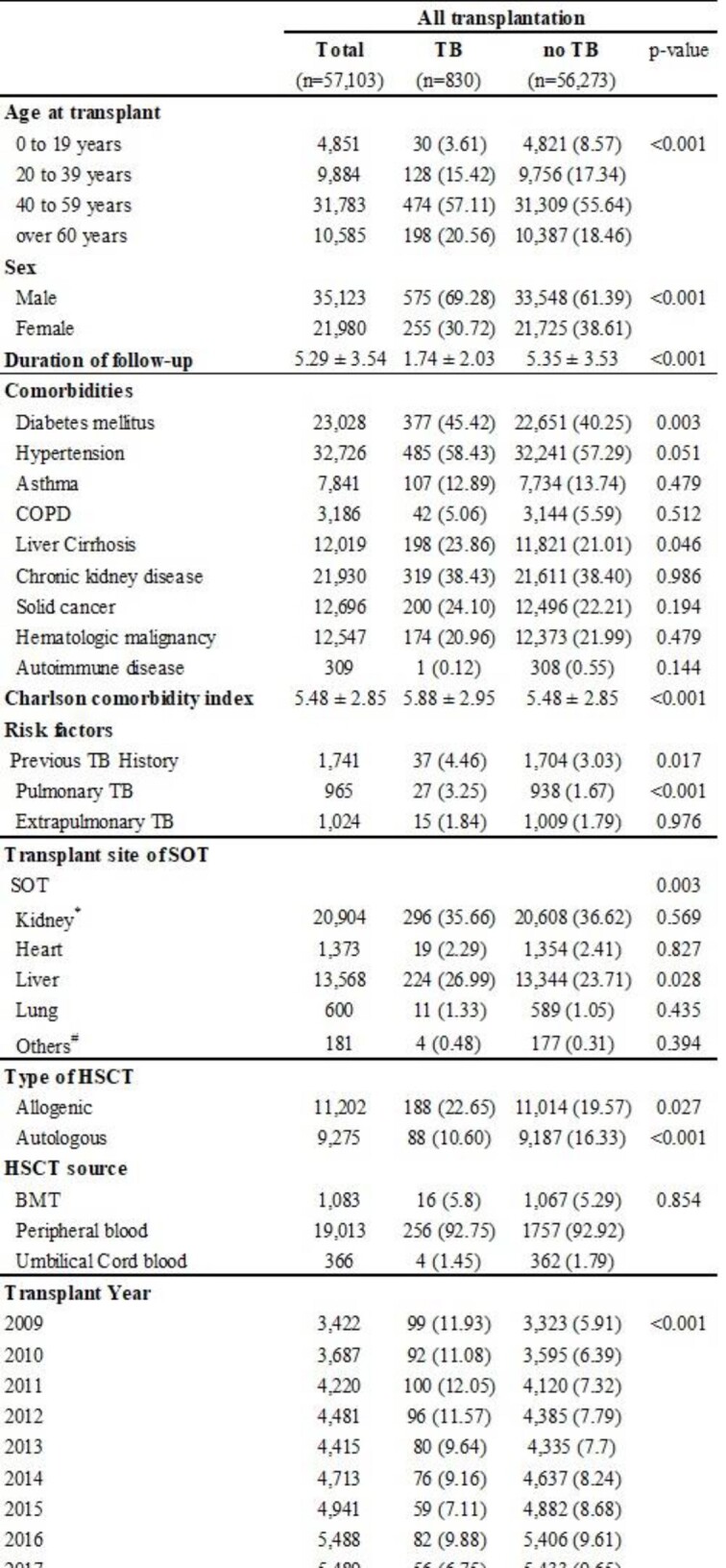

Cumulative incidence of TB following transplant

**Figure:**
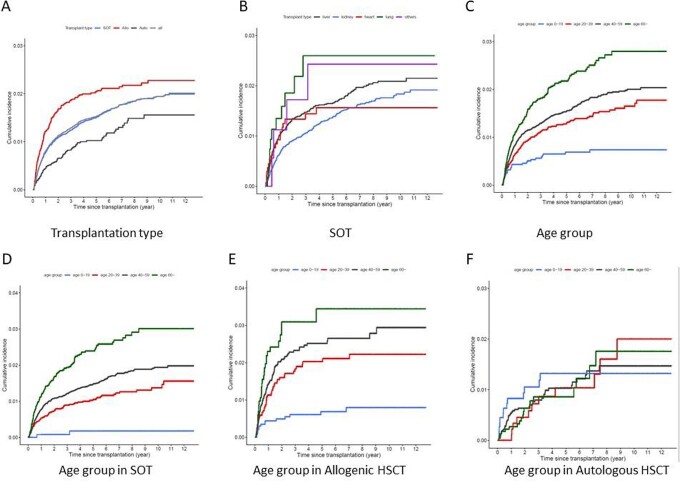

Risk factor of tuberculosis after transplanatation

**Figure:**
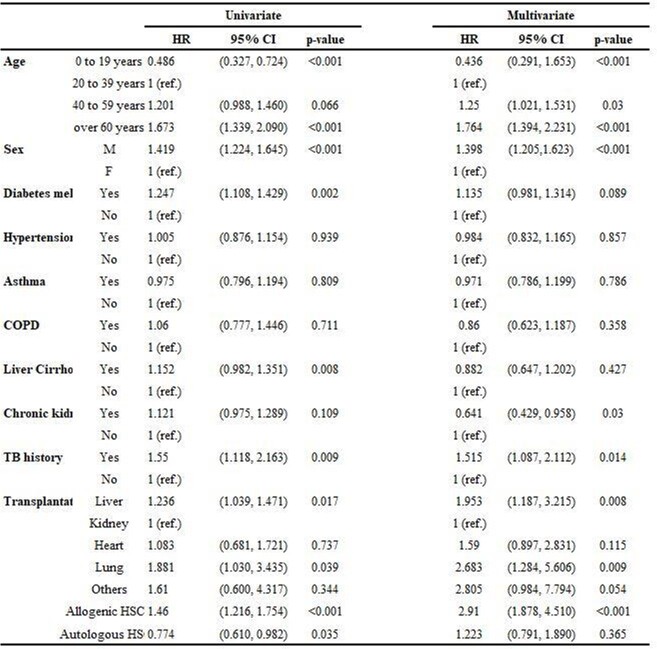

**Conclusion:**

The incidence of TB in transplant recipient is at least three times higher than the general population in South Korea. Regardless of the type of transplant, TB should be considered as a complication within 1 years of transplantation and special attention should be paid to patients with a history of previous TB history.

**Disclosures:**

**All Authors**: No reported disclosures.

